# Research on Disaster Environment Map Fusion Construction and Reinforcement Learning Navigation Technology Based on Air–Ground Collaborative Multi-Heterogeneous Robot Systems

**DOI:** 10.3390/s25164988

**Published:** 2025-08-12

**Authors:** Hongtao Tao, Wen Zhao, Li Zhao, Junlong Wang

**Affiliations:** 1 School of Mechanical Engineering, University of Science and Technology Beijing, Beijing 100083, China; 2 School of Civil Aviation, Northwestern Polytechnical University, Xi’an 710072, China; 3 Yunnan Key Laboratory of Unmanned Autonomous Systems, Yunnan Minzu University, Kunming 650500, China; zhaoli@ymu.edu.cn; 4 Faculty of Science and Technology, Waseda University, Tokyo 169-8555, Japan

**Keywords:** disaster map construction, deep reinforcement learning, path planning, high-precision map fusion

## Abstract

The primary challenge that robots face in disaster rescue is to precisely and efficiently construct disaster maps and achieve autonomous navigation. This paper proposes a method for air–ground collaborative map construction. It utilizes the flight capability of an unmanned aerial vehicle (UAV) to achieve rapid three-dimensional space coverage and complex terrain crossing for rapid and efficient map construction. Meanwhile, it utilizes the stable operation capability of an unmanned ground vehicle (UGV) and the ground detail survey capability to achieve precise map construction. The maps constructed by the two are accurately integrated to obtain precise disaster environment maps. Among them, the map construction and positioning technology is based on the FAST LiDAR–inertial odometry 2 (FAST-LIO2) framework, enabling the robot to achieve precise positioning even in complex environments, thereby obtaining more accurate point cloud maps. Before conducting map fusion, the point cloud is preprocessed first to reduce the density of the point cloud and also minimize the interference of noise and outliers. Subsequently, the coarse and fine registrations of the point clouds are carried out in sequence. The coarse registration is used to reduce the initial pose difference of the two point clouds, which is conducive to the subsequent rapid and efficient fine registration. The coarse registration uses the improved sample consensus initial alignment (SAC-IA) algorithm, which significantly reduces the registration time compared with the traditional SAC-IA algorithm. The precise registration uses the voxelized generalized iterative closest point (VGICP) algorithm. It has a faster registration speed compared with the generalized iterative closest point (GICP) algorithm while ensuring accuracy. In reinforcement learning navigation, we adopted the deep deterministic policy gradient (DDPG) path planning algorithm. Compared with the deep Q-network (DQN) algorithm and the A* algorithm, the DDPG algorithm is more conducive to the robot choosing a better route in a complex and unknown environment, and at the same time, the motion trajectory is smoother. This paper adopts Gazebo simulation. Compared with physical robot operation, it provides a safe, controllable, and cost-effective environment, supports efficient large-scale experiments and algorithm debugging, and also supports flexible sensor simulation and automated verification, thereby optimizing the overall testing process.

## 1. Introduction

With the development of technology, the application of robots has become increasingly common in our daily lives, such as in simple services, and even in the fields of Military [1] and disaster rescue [2]. In disaster rescue, air–ground collaborative multi-heterogeneous robotic systems can overcome the limitations of single-robot systems [3]. By combining UGV and UAV, air–ground collaborative systems are better able to adapt to the complexity of disaster environments. UAVs can quickly perform wide-area reconnaissance, providing environmental information from a global perspective, while UGVs can venture into hazardous areas or narrow spaces to carry out precise operations and task executions. This air–ground collaborative mode allows robots to adapt flexibly in dynamic environments, significantly enhancing the accuracy and scope of environmental perception. In disaster scenarios, if one robot encounters a failure or is interfered with by the environment, other robots can still continue their tasks, ensuring that rescue operations are not significantly disrupted. Therefore, air–ground collaborative multi-heterogeneous robots have broad application potential in disaster rescue. Therefore, this paper proposes an innovative air–ground collaborative multi-heterogeneous robotic system, which integrates advanced mapping, map fusion, and autonomous navigation technologies. This approach enables more accurate map surveying, thereby enhancing the effectiveness of robot-assisted rescue operations.

In robotic technologies, simultaneous localization and mapping (SLAM) has emerged as a core technology for autonomous navigation, receiving widespread attention in both research and applications in recent years. SLAM enables robots to construct maps and perform self-localization in unknown environments by utilizing sensor data, providing a solid foundation for precise navigation [4]. However, traditional SLAM systems often rely on a single type of sensor or robotic platform, which introduces certain limitations [5]. In disaster environments, a single sensor may struggle to cope with the complexity and dynamic nature of the surroundings. To address these challenges, this paper applies the FAST-LIO2 framework, which integrates multi-sensor fusion to enhance the accuracy and robustness of the mapping process. Compared with traditional SLAM, FAST-LIO2 has a smaller absolute position error (APE), which is one of the criteria for mapping. This is because FAST-LIO2 tightly couples the inertial odometer and the LiDAR, enabling it to predict the pose of the LiDAR at any moment in a frame during backpropagation, thereby achieving motion distortion compensation [6].

After obtaining two environment point cloud maps with distinct advantages using FAST-LIO2, a comprehensive study of the air–ground map fusion algorithm is conducted, incorporating various steps such as point cloud preprocessing, coarse registration, and fine registration. To address the issues of slow convergence in the traditional SAC-IA coarse registration algorithm [7], an improved SAC-IA algorithm is applied [8]. Through simulation experiments, it can be seen that the improved SAC-IA algorithm reduces the time by more than 50% compared to the traditional SAC-IA algorithm, which is similar to that of Zhang Han et al. Furthermore, to tackle the high computational cost and low efficiency encountered in the traditional GICP algorithm when processing large-scale point cloud data, a modified VGICP algorithm is introduced [9]. This algorithm leverages voxelization techniques, which eliminates the need for the expensive nearest-neighbor search inherent in traditional methods, significantly improving computational efficiency.

In disaster rescue tasks, the primary advantage of DDPG over DQN lies in its ability to handle continuous action spaces, enabling more precise control and task execution for the robot [10,11]. DDPG utilizes noise injection for exploration, overcoming the limitations of discrete actions in DQN, thus providing a more stable and efficient learning process. Additionally, DDPG employs soft updates for the target network, enhancing training stability and enabling fine-grained decision-making in complex, dynamic disaster environments. Guo Siyu et al. enabled the unmanned ship to achieve autonomous path planning through DDPG, and also verified that DDPG has a faster convergence speed and better decision-making ability compared to DQN [12].

Compared to traditional disaster rescue robots, the air–ground collaborative disaster rescue robot proposed in this paper offers several innovations. Its air–ground collaborative working mode allows the drone and unmanned ground vehicle to complement each other’s strengths, enabling coverage of both extensive areas and complex terrains, thereby improving operational efficiency and task completion. Additionally, the use of reinforcement learning for path planning provides the robot with the ability to dynamically adjust its path in real time. This capability allows the robot to flexibly optimize its action strategy in response to changes in the disaster environment, leading to more efficient and precise task execution in uncertain conditions.

All of the above algorithm simulations were developed based on the ROS 2 framework. Point cloud processing relied on the Open3D library, reinforcement learning path planning relied on the PyTorch 2.0 library, and they were deployed on the Ubuntu 20.04 system. At the same time, the design process of some algorithms will also be presented in the following text.

## 2. Materials and Methods

### 2.1. FAST-LIO2 Framework

FAST-LIO2 is a computationally efficient LiDAR–inertial odometry (LIO) system based on tightly-coupled iterative Kalman filtering, proposed by Wei Xu et al [6]. Its state estimation module integrates a multi-stage pipeline encompassing IMU-LiDAR data fusion, motion prediction, motion distortion compensation, residual propagation and correction, and incremental state updates, ultimately delivering robust state estimation for real-time SLAM systems.

**Figure 1:**
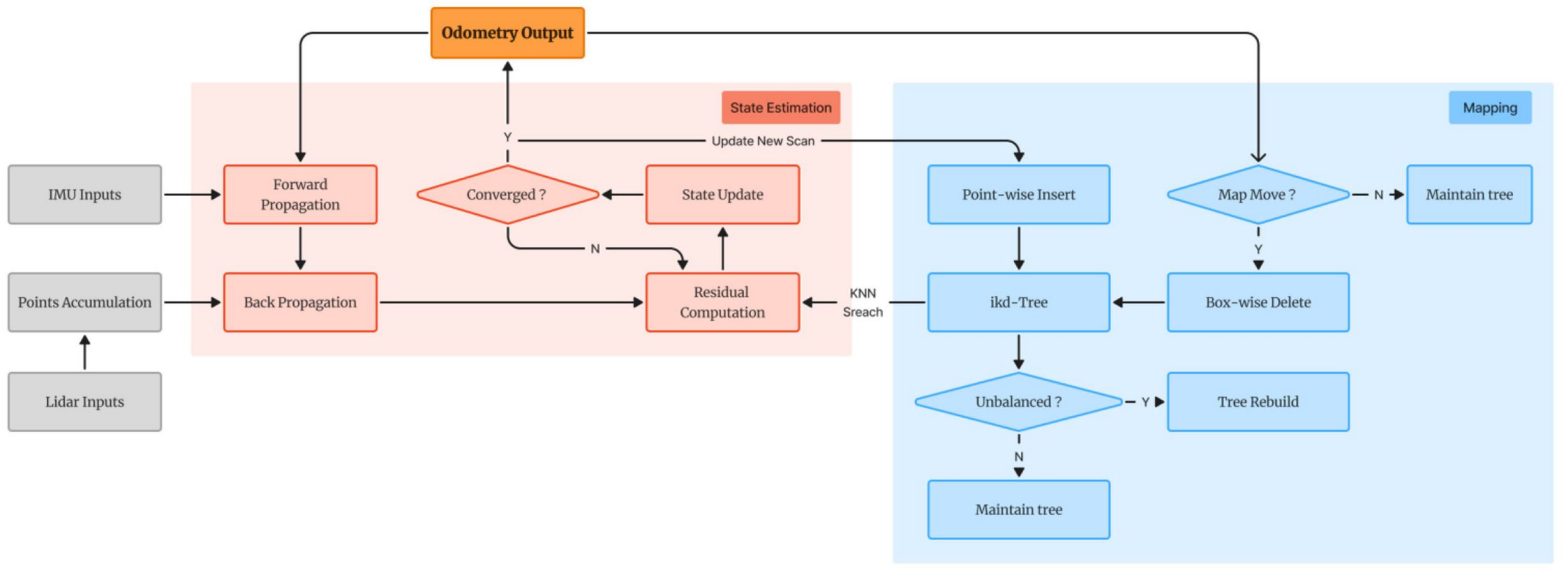
System of FAST-LIO2.

FAST-LIO2 introduces two key innovations. Firstly, it aligns the raw point clouds directly with the map, eliminating the need for feature extraction and instead using all point cloud data. This approach fully utilizes the subtle features in the environment, enhances the registration accuracy, and saves computational resources and time by omitting additional feature extraction steps, enabling the system to operate more efficiently in real-time. Secondly, it employs an incremental kd-Tree structure (ikd-Tree). The traditional kd-Tree typically requires rebuilding the entire tree with each new point insertion or deletion, which demands significant computational resources and time [13]. The ikd-Tree adopts an incremental updating approach, allowing gradual updates to the tree structure as new point cloud data is continually received, without the need for complete reconstruction, thus avoiding the waste of computational resources in traditional spatial data structures when dealing with large-scale and dynamic data. The system architecture of FAST-LIO2 is shown in Figure 1 [6], which is mainly divided into the state estimation module and the mapping module.

#### 2.1.1. Forward Propagation

Forward propagation predicts the current pose and velocity of the system based on the acceleration and angular velocity from the IMU. This process begins with the state estimation from the previous time step, and updates the state using the current IMU observations. The predicted state from the IMU provides an initial estimate for the next time step, which is then used as the initial value for the LiDAR data optimization. Through forward propagation, the system can rapidly update the robot’s position and orientation, reducing the reliance on LiDAR data and enhancing both computational efficiency and real-time performance. Forward propagation provides a good initial estimate for LiDAR optimization, and the point cloud information provided by the LiDAR further refines the result of the forward propagation, thereby enabling the fusion of IMU and LiDAR data.

#### 2.1.2. Back Propagation

Backward propagation begins by comparing the state estimates obtained from forward propagation with the actual LiDAR data and map to calculate the residuals. A residual function is then constructed, and the residual function is minimized using an optimization algorithm (Gaussian–Newton method). Through iterative optimization, the algorithm adjusts the estimates based on the results from each iteration until the residual function converges, ultimately yielding an accurate state estimate. During LiDAR data acquisition, point clouds are accumulated over each scanning cycle. Consequently, different feature points are scanned at different times, and if the robot is in motion during this phase, its reference frame also moves. The scene observed by the LiDAR is affected by this motion, leading to motion distortions that impact the accuracy of the data. In backward propagation, the IMU-provided acceleration and angular velocity data are used to estimate and compensate for the motion distortions in the LiDAR data [14].

#### 2.1.3. Incremental Update and Rebalancing of ikd-Tree

In FAST-LIO2, incremental updates to the kd-tree are primarily achieved through point insertion and box deletion operations. Point insertion utilizes a tree downsampling algorithm. Initially, based on the estimated state and the given resolution *l*, the space is divided into cubes with side length *l*, and the cube *C* containing the point *P* is identified. Within *C*, the point $P_{nearest}$, which is closest to the center $P_{center}$, is located, and all other points are deleted. The point $P_{nearest}$ is then inserted into the ikd-tree. The insertion process begins with a recursive search from the root node, comparing the point in question with the tree nodes, recursively partitioning along the axis until an empty node is found, at which point the insertion point is stored and a new leaf node is created with initialized properties. Following the insertion, balance checks are performed to ensure the tree’s structural integrity.

Box deletion is carried out using lazy tags and range information, specifically the states of “deleted” and “treedeleted” in the node structure. Points marked as “deleted” are not immediately removed; instead, they are deleted during the tree reconstruction process. When all child nodes of a root node are marked as “deleted”, the root node itself is tagged as “treedeleted”. In the box deletion operation, if the bounding box CO containing the points to be deleted does not intersect with the bounding box CT, no update occurs. If CT completely contains CO, the states of “deleted” and “treedeleted” are set to true. If CT intersects with CO and the point *P* is within CO, point *P* is deleted, followed by attribute updates and rebalancing.

The balancing criteria for the ikd-tree consist of the α-balancing criterion and the α-deletion criterion. For a node *T* and its children, the α-balancing criterion is satisfied when:

(1) $$\begin{array}{c} S\left(T.leftchild\right)<\alpha_{bal}\left(S\left(T\right)-1\right)\\ S\left(T.rightchild\right)<\alpha_{bal}\left(S\left(T\right)-1\right) \end{array}$$

where S(T) represents the treesize attribute of node *T*, and $\alpha_{bal}$ is a balancing factor for the tree, with $\alpha_{bal}\in \left(0.5,1.0\right)$. This condition limits the number of points in the left and right subtrees, thereby controlling the height of the tree. When the following conditions are met, the tree satisfies the α-deletion criterion:

(2) $$I\left(T\right)<\alpha_{del}S\left(T\right)$$

where I(T) denotes the number of ineffective points in node *T*, and $\alpha_{del}$ is the deletion factor that controls the number of ineffective points to be removed, with $\alpha_{del}\in \left(0,1\right)$. This condition ensures that ineffective points are removed from the tree without disturbing its structure. The tree is considered balanced when both conditions in Equations (1) and (2) are satisfied. If any node fails to meet these criteria, a rebalancing operation is performed to restore the tree’s balance.

#### 2.1.4. Tree Rebuild

**Figure 2:**
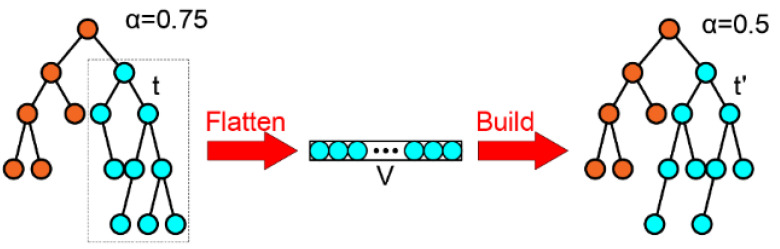
Reconstructing the unbalanced Subtree.

When the reconstruction scale is large and time becomes a bottleneck, the ikd-tree may be monopolized. To address this, a dual-thread reconstruction method is used to improve system efficiency. When the tree’s size is smaller than the threshold $N_{max}$, reconstruction is performed using the main thread. Otherwise, reconstruction is handled by a secondary thread to ensure a balanced tree. During this process, insertions are temporarily suspended, and updates are stored in a vector V of active points from child nodes. The balance increment updates are logged into the OperationLogger. After completing all requests, the new balanced subtree $t^{\prime }$ replaces the original subtree *t*. Specifically, when the balance criterion detects that a certain subtree is unbalanced, all the nodes of the subtree will be randomly rearranged. After removing the nodes that have been marked for deletion, the remaining nodes will be reconstructed and then replaced back to their original positions, as shown in Figure 2. If the balance criterion determines that this subtree is balanced, the the tree is kept in its original form.

### 2.2. Preprocess

**Figure 3:**
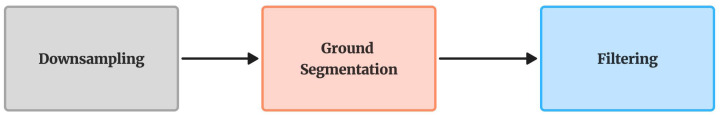
Process of point cloud preprocessing.

To enhance the quality and efficiency of the original point cloud data, as well as to provide a streamlined and reliable dataset for subsequent registration processes [15], a point cloud preprocessing workflow is illustrated in Figure 3. Initially, point cloud downsampling is performed, which reduces the number of data points, thereby lowering computational load and storage requirements while preserving the principal geometric features. This is followed by point cloud segmentation, which divides complex point cloud data into independent regions or objects with similar characteristics, thus providing a more precise and efficient data foundation for subsequent target recognition, scene reconstruction, and feature extraction. Finally, point cloud filtering is conducted to remove noise, outliers, and redundant data, while retaining critical geometric information, thereby reducing data volume and computational complexity.

In this paper, we employ voxel downsampling, a commonly used method for simplifying point cloud data. The fundamental concept involves partitioning the three-dimensional space into a series of equally-sized voxels (cubes), and replacing all points within each voxel with a representative point, typically the centroid. The aim of voxel downsampling is to transform the original point cloud $P=\left\{p_{i}=\left(x_{i},y_{i},z_{i}\right)\in R^{3}|i=1,i=2,\ldots ,N\right\}$ into a new point cloud $P_{down}$, which has fewer points but still approximately preserves the overall structure of the original cloud. We begin by defining a voxel as a cube with edges $\Delta_{x}=\Delta_{y}=\Delta_{z}=a$. The index $v_{i}$ of the voxel unit to which each point $p_{i}$ in point cloud *P* belongs can be calculated by

(3) $$v_{i}=\left(\left\lfloor \frac{x_{i}}{\Delta_{x}}\right\rfloor ,\left\lfloor \frac{y_{i}}{\Delta_{y}}\right\rfloor ,\left\lfloor \frac{z_{i}}{\Delta_{z}}\right\rfloor \right)$$

where notation ⌊·⌋ represents the floor function, which rounds down to the nearest integer. The term $v_{i}$ can be understood as the voxel containing the point $p_{i}$. Thus, the set of points contained within a voxel unit $V_{k}$ can be determined as

(4) $$P_{V_{k}}=\left\{p_{i}\in P|v_{i}=V_{k}\right\}$$

In the voxel unit $V_{k}$, all points are replaced by the centroid $c_{\left(V_{k}\right)}$ of the points contained within. The formula for calculating the centroid is as follows:

(5) $$c_{V_{k}}=\frac{1}{n_{V_{k}}}\sum_{p\in P_{V_{k}}}x,\frac{1}{n_{V_{k}}}\sum_{p\in P_{V_{k}}}y,\frac{1}{n_{V_{k}}}\sum_{p\in P_{V_{k}}}z$$

The ground plane fitting (GPF) algorithm is well suited for applications such as autonomous driving and robotic navigation, where large-scale LiDAR point cloud data must be processed in real time [16]. Its key advantages include fast computation, a simple structure, stable iterative convergence, and a deterministic approach that avoids random sampling. GPF effectively filters out ground points, providing a reliable foundation for subsequent obstacle detection and scene understanding. In this work, the GPF algorithm is adopted for ground segmentation.

To enhance the robustness of plane fitting, GPF introduces the concept of the lowest point representative (LPR). The algorithm begins by sorting the input point cloud *P* in ascending order based on height. It then selects the lowest $N_{L}PR$ points and computes their average height as follows:

(6) $$h_{LPR}=\frac{1}{N_{LPR}}\sum_{i=1}^{N_{LPR}}z_{i}$$

where $z_{i}$ denotes the height coordinate of a point. A threshold $Th_{seeds}$ is then applied to ensure that the selected seed points are sufficiently close to the actual ground surface. Specifically, all points satisfying $z\leq h_{LPR}+Th_{seeds}$ are selected to form the initial ground seed set S. Assuming the ground surface can be approximated by a planar model, the mathematical representation of the plane is given by:

(7) $$n^{T}x+d=0$$

where n denotes the normal vector of the plane, and x represents the coordinates of a point in 3D space. To estimate the plane parameters, a set of seed points S is first selected. The covariance matrix $C\in R^{\left(3\times 3\right)}$ of the seed points is then computed as:

(8) $$C=\sum_{i=1}^{|S|}\left(s_{i}-\bar{s}\right){\left(s_{i}-\bar{s}\right)}^{T}$$

where $\bar{s}$ denote the mean of the seed point set. The covariance matrix C is then decomposed using singular value decomposition (SVD), yielding a set of eigenvectors. The eigenvector corresponding to the smallest eigenvalue represents the normal vector n of the estimated ground plane. Subsequently, the plane offset $d=-n^{T}\bar{s}$ can be computed, allowing the distance from any point $p_{k}$ to the fitted plane be expressed as:

(9) $$D_{k}=|n^{T}p_{k}+d|$$

A threshold $Th_{dist}$ is set for the point-to-plane distance. If the distance $D_{k}$ of point $p_{k}$ to the plane satisfies $D_{k}<Th_{dist}$, the point is classified as a ground point. Otherwise, it is labeled as a non-ground point. The newly identified ground points are then used to update the seed set, and the above process is repeated for $N_{iter}$ iterations to iteratively refine the plane model until convergence.

Statistical filtering is a critical step in point cloud preprocessing, aiming to effectively eliminate noise by analyzing the geometric distribution within local neighborhoods [17,18]. The core principle is grounded in the assumption of a Gaussian distribution: Points that are consistent with the local surface typically exhibit neighborhood distances concentrated around a mean value, while outliers deviate significantly from this pattern. This section presents a detailed description of the statistical filtering model, algorithmic workflow, and parameter optimization strategies.

Let *P* denote the original point cloud. For a point $p_{i}\in P$, its local neighborhood $N_{i}$ is defined by its *k*-nearest neighbors. The Euclidean distances from $p_{i}$ to all points in $N_{i}$ are then computed using the standard formula:

(10) $$\begin{array}{cc} d_{ij} & =∥p_{i}-p_{j}∥\\ \; & =\sqrt{{\left(x_{i}-x_{j}\right)}^{2}+{\left(y_{i}-y_{j}\right)}^{2}+{\left(z_{i}-z_{j}\right)}^{2}} \end{array}$$

where $p_{j}$ is a point in the neighborhood $N_{i}$. Subsequently, the neighborhood statistics are computed by evaluating the mean $\mu_{i}$ and standard deviation $\sigma_{i}$ of the Euclidean distances from each point to its neighboring points, as defined by:

(11) $$\begin{array}{cc} \mu_{i} & =\frac{1}{k}\sum_{j=1}^{k}d_{ij}\\ \sigma_{i} & =\sqrt{\frac{1}{k}\sum_{j=1}^{k}{\left(d_{ij}-\mu_{i}\right)}^{2}} \end{array}$$

where $\mu_{i}$ represents the average neighborhood density for point *i*, while $\sigma_{i}$ characterizes the degree of dispersion within the local neighborhood. A global statistical threshold is then established by computing the overall mean $\mu_{global}$ and standard deviation $\sigma_{global}$ of these local mean distances across all data points, as defined by:

(12) $$\begin{array}{cc} \mu_{global} & =\frac{1}{N}\sum_{i=1}^{N}\mu_{i}\\ \sigma_{global} & =\sqrt{\frac{1}{N}\sum_{i=1}^{N}{\left(\mu_{i}-\mu_{global}\right)}^{2}} \end{array}$$

The outlier rejection threshold is defined as $T=\mu_{global}+\alpha \sigma_{global}$. A point is classified as an outlier and subsequently removed if its average neighborhood distance $\mu_{i}$ exceeds the threshold *T*. The parameter α is an empirical constant that controls the strictness of the filtering process. A larger value of α results in a more conservative filter, retaining most points and removing only those with significantly large deviations, whereas a smaller α leads to a more aggressive filter, eliminating a greater number of potential outliers.

### 2.3. Coarse Registration

After point cloud preprocessing, it becomes feasible to extract geometrically consistent and structured representations from low-quality raw data. However, the core challenge of point cloud registration lies in estimating the rigid transformation between two point sets, especially in scenarios where the initial pose is completely unknown. Traditional single-stage optimization methods, such as the iterative closest point (ICP) algorithm, are prone to local minima due to their sensitivity to initial alignment. To address this, a hierarchical registration strategy is typically adopted: coarse registration performs a global search for plausible alignment hypotheses, while fine registration refines the transformation locally based on the initial alignment [19]. In the following, we present a detailed investigation of a coarse registration method based on an improved sample consensus initial alignment (SAC-IA), followed by fine registration using the VGICP algorithm.

In this study, we adopt the improved sample consensus initial alignment (SAC-IA) algorithm proposed by Zhang et al [7]. Building upon the conventional SAC-IA framework, this method introduces scanning angle constraints and geometric shape filtering to effectively reduce the randomness and collinearity issues in feature point selection, thereby enhancing the stability of feature matching. Furthermore, the algorithm employs a three-part error evaluation strategy based on differences in FPFH descriptors [20], Euclidean distances [21], and geometric slopes [22]. This enhances the ability to detect and correct mismatches, avoids convergence to local minima, and reduces the time of the algorithm. The transformation matrix is accepted only when it meets the threshold conditions for initial rotation and translation, thus providing a more accurate initialization for subsequent fine registration. The improved algorithm significantly outperforms the traditional SAC-IA method in terms of computational efficiency, particularly in scenarios involving sparse point clouds or substantial feature variation.

#### 2.3.1. Feature Point Selection in the Target Point Cloud

In the conventional SAC-IA algorithm, the randomness in feature point selection often leads to mismatches due to incorrectly or poorly selected feature points. To address this issue, the improved SAC-IA algorithm introduces constraints on both the scanning angle of the feature set and the projection shape of feature points. Regarding the scanning angle constraint, it is assumed that for a point P(x,y,z) in the point cloud, the elevation angle ω between the point and the 2D-plane can be calculated as follows:

(13) $$\omega =\arctan(\frac{z}{\sqrt{x^{2}+y^{2}}})$$

Next, the scanning angle $s_{i}$ is defined, and the set of scanning angles $s_{A}$ is denoted as:

(14) $$s_{A}=\left\{0^{{}^{\circ}},{\pm 22.5}^{{}^{\circ}},{\pm 45}^{{}^{\circ}},{\pm 67.5}^{{}^{\circ}},{\pm 90}^{{}^{\circ}},{\pm 112.5}^{{}^{\circ}},{\pm 135}^{{}^{\circ}},{\pm 157.5}^{{}^{\circ}},{\pm 180}^{{}^{\circ}}\right\}$$

The elevation angle range is divided into several intervals corresponding to the elements in $s_{A}$. For each point P(x,y,z) in the point cloud, its elevation angle is compared with each $s_{i}$ in the set. If $|\omega -s_{i}|\leq A$, the point is considered to correspond to $s_{i}$ and is selected as a candidate feature point, where *A* is a threshold for the allowable difference between the elevation angle and the scanning angle.

To avoid selecting points that are overly concentrated or overly sparse, and to impose constraints on the spatial projection of feature points, one candidate point is randomly selected from the group corresponding to $s_{i}=0^{{}^{\circ}}$, denoted as $P_{0}\left(x_{0},y_{0},z_{0}\right)$. Then, two additional points are selected from the candidate groups corresponding to $s_{i}=\pm 22.5^{{}^{\circ}}$, satisfying:

(15) $$D_{H}>y_{1}-y_{0}>D_{L}$$

where $D_{H}$ and $D_{L}$ are the upper and lower bounds for the vertical (*y*-axis) spacing of the selected points, respectively. The process is repeated for $s_{i}=\pm 45^{{}^{\circ}}$, and so on, to iteratively construct a set of feature points.

The projection of this feature point set onto the ωoY plane is constrained to form an approximate triangle, which effectively mitigates the issues of point degeneracy along the same direction and local point cloud sparsity.

#### 2.3.2. Calculation of FPFH Values

The FPFH algorithm introduces the concept of the simplified point feature histogram (SPFH), which computes geometric relationships only between each point and its neighbors [6]. Compared to PFH, this reduces the computational complexity significantly. The SPFH is first computed by evaluating a set of angular features between the source point $p_{q}$ and its neighbor $p_{k}$ using the following expressions:

(16) $$\begin{array}{cc} \alpha & =v\cdot n_{k}\\ \phi & =\left(u\cdot \left(p_{k}-p_{q}\right)\right)/∥p_{k}-p_{q}∥\\ \theta & =\arctan(w\cdot n_{k},u\cdot n_{k}) \end{array}$$

where the three orthogonal basis vectors are defined as $u=n_{q},v=\left(p_{k}-p_{q}\right)\times u,w=u\times v$. α measures the angle between the neighbor’s normal vector $n_{k}$ and the direction vector from $p_{q}$ to $p_{k}$. ϕ represents the angle between the vector *u* and the line segment connecting $p_{q}$ and $p_{k}$. θ captures the angular difference between the normal vectors of $p_{q}$ and $p_{k}$.

Once the SPFH is calculated for each point, the FPFH descriptor of the source point $p_{q}$ is computed by aggregating its own SPFH and the weighted SPFH contributions from its neighbors, as given by:

(17) $$FPFH\left(p_{q}\right)=SPF\left(p\cdot p_{q}\right)+\frac{1}{k}\sum_{i=1}^{k}\frac{1}{\omega_{k}}\cdot SPF\left(p_{k}\right)$$

#### 2.3.3. Computation of Transformation Matrix

To estimate the transformation between the source and target point clouds, point pairs $p_{i}$ and $q_{i}$ with similar FPFH descriptors are first selected. The rotation matrix and translation vector are then computed using singular value decomposition (SVD). The covariance matrix *H* between the source and target point sets is constructed as:

(18) $$H=\sum_{i=1}^{n}\left(p_{i}-\bar{p}\right){\left(q_{i}-\bar{q}\right)}^{T},H=U\cdot \Sigma \cdot V^{T}$$

where $\bar{p}$ and $\bar{q}$ represent the centroids of all source and target points, respectively. *U* and *V* are 3×3 orthogonal matrices, and Σ is the diagonal matrix of singular values of *H*. The rotation matrix *R* is then computed by:

(19) $$R=V\cdot U^{T}$$

Once the rotation matrix *R* is obtained, the translation vector *t* can be derived to complete the rigid alignment. It is calculated based on the centroid difference between the point sets:

(20) $$t=\bar{q}-R\cdot \bar{p}$$

And the full transformation matrix *T* is expressed as:

(21) $$T=\left[\begin{array}{cc} R & t\\ 0 & 1 \end{array}\right]$$

#### 2.3.4. Mismatch Correction

In point cloud registration, mismatches are inevitable, especially when the point cloud contains noise or when feature point selection is suboptimal. The presence of mismatches can lead to inaccurate registration results, thereby affecting the subsequent fine registration process. To address this issue, the improved SAC-IA algorithm incorporates a mismatch correction mechanism. In this process, three evaluation criteria are introduced: FPFH histogram difference, Euclidean distance deviation, and geometric slope deviation. After initial registration, for each matched point pair, the differences in their FPFH descriptors, Euclidean distances, and geometric slopes are calculated. If all three deviations are below their respective thresholds, the match is considered valid and the algorithm proceeds to the next step. Otherwise, the target feature point in the point cloud is reselected.

### 2.4. Fine Registration

After performing coarse registration between the source and target point clouds, an initial alignment is obtained. However, due to factors such as noise, low overlap ratio, or large initial pose deviation, only a sub-optimal alignment may be achieved. Therefore, fine registration is required to further refine the alignment. Based on the initial pose provided by the coarse registration, the algorithm computes the nearest neighbor correspondences and minimizes the distance errors to eliminate local misalignment. The VGICP algorithm, which replaces traditional nearest neighbor search with a voxel-based definition of closest points, avoids expensive computations, significantly improves efficiency and robustness, and is well-suited for real-time SLAM applications. Accordingly, this paper adopts the VGICP algorithm to achieve fine registration between the source and target point clouds.

In the GICP algorithm, suppose the source point cloud is $A=\left\{a_{0},a_{1},\ldots ,a_{N}\right\}$ and the target point cloud is $B=\left\{b_{0},b_{1},\ldots ,b_{M}\right\}$, with the goal of estimating the transformation matrix T between them. It is assumed that each point follows a Gaussian distribution, namely, $a_{i}∼N\left(\hat{a_{i}},C_{A}\right),b_{i}∼N\left(\hat{b_{i}},C_{B}\right)$, where $\hat{a_{i}}$ and $\hat{b_{i}}$ are the mean values, and $C_{A}$ and $C_{B}$ are the corresponding covariance matrices. The transformation error between the corresponding points $a_{i}$ and $b_{i}$ is defined as:

(22) $$\hat{d_{i}}=\hat{b_{i}}-T\hat{a_{i}}$$

According to the properties of Gaussian error propagation, the distribution of $d_{i}$ is given by:

(23) $$d_{i}∼N\left(0,C_{B}+TC_{A}T^{T}\right)$$

To solve for the optimal transformation matrix, GICP employs the maximum likelihood estimation:

(24) $$T=\arg\min_{T}\sum_{i}d_{i}^{T}{\left(C_{B}+TC_{A}T^{T}\right)}^{-1}d_{i}$$

Unlike traditional GICP, the VGICP algorithm avoids reliance on precise nearest neighbor search by utilizing voxel-based distribution comparison. First, the distribution model is extended to multiple neighboring points. For each $a_{i}$ and its neighborhood $\left\{b_{j}|∥a_{i}-b_{j}∥<r\right\}$, the error term is defined as:

(25) $$\hat{d_{i}^{\prime }}=\sum_{j}\left(\hat{b_{j}}-T\hat{a_{i}}\right)$$

This can be viewed as a smoothing process over the target distribution. Similar to GICP, the mean and covariance of the transformed error distribution are:

(26) $$\begin{array}{cc} \mu_{d_{i}} & =\sum_{j}\left(\hat{b_{j}}-T\hat{a_{i}}\right)=0,\\ C_{d_{i}} & =\sum_{j}\left(C_{B_{j}}+TC_{A_{i}}T^{T}\right) \end{array}$$

Then, the transformation matrix *T* is estimated via maximum likelihood as:

(27) $$T=\arg\min_{T}\sum_{i}{d_{i}^{\prime }}^{T}C_{d_{i}}^{-1}d_{i}^{\prime }$$

The estimated transformation matrix is applied iteratively to update the source point cloud. This process continues until the transformation error is smaller than a predefined threshold or convergence is achieved, yielding the optimal transformation matrix.

### 2.5. DDPG-Based Reinforcement Learning Path Planning

The deep deterministic policy gradient (DDPG) algorithm improves upon DQN primarily by enabling the effective handling of continuous action spaces [10]. It adopts a deterministic policy and leverages the Actor–Critic architecture. Unlike DQN, which operates in discrete action spaces and relies on the ϵ-greedy strategy, DDPG employs an actor network to output continuous deterministic actions and a critic network to evaluate these actions by estimating their Q-values.

**Figure 4:**
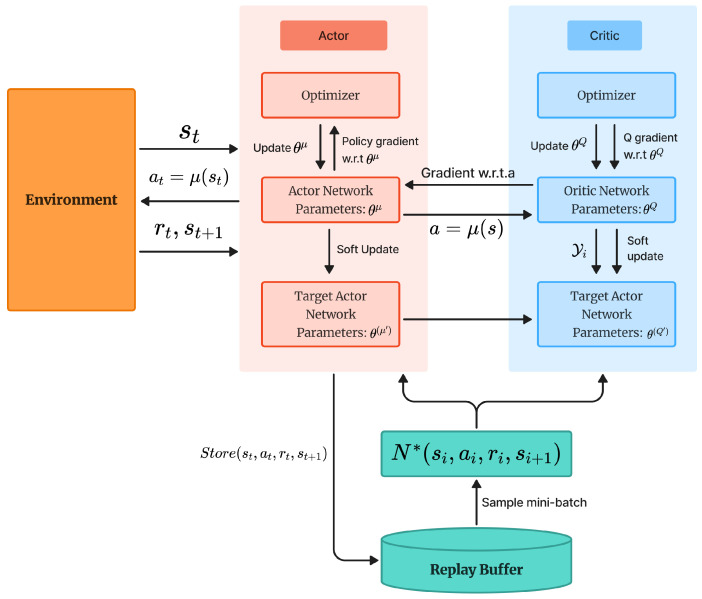
Process of DDPG.

Moreover, DDPG inherits the experience replay and target network mechanisms from DQN. To encourage exploration, noise is added to the deterministic actions, enabling stochastic behavior in continuous action spaces. Additionally, DDPG uses a soft update mechanism to gradually update the target networks, which contributes to improved training stability. The overall workflow of DDPG is illustrated in Figure 4.

During the DDPG process, the Actor and Critic networks are first initialized similarly to DQN, with the Actor network containing policy network parameters $\theta^{\mu }$, and the Critic network containing Q-network parameters $\theta^{Q}$, along with their corresponding target networks $\theta^{\left(\mu^{\prime }\right)}$ and $\theta^{\left(Q^{\prime }\right)}$. The target networks are updated softly to track the parameters of their respective networks.

The Actor network takes the current state $s_{t}$ as input and outputs a deterministic action $a_{t}=\mu \left(s_{t}|\theta^{\mu }\right)$. To encourage exploration, noise is added to the action. The interaction tuple $\left(s_{t},a_{t},r_{t},s_{\left(t+1\right)}\right)$ is then stored in the replay buffer. During training, a minibatch of experiences $\left(s_{i},a_{i},r_{i},s_{\left(i+1\right)}\right)$ is sampled for network updates.

Within the Critic network framework, the target Q-value is computed as follows:

(28) $$y_{i}=r_{i}+\gamma Q^{\prime }(s_{i+1},\mu^{\prime }(s_{i+1}|\theta^{\mu^{\prime }})|\theta^{Q^{\prime }})$$

where γ is the discount factor, $Q^{\prime }$ denotes the target Critic network, and $\mu^{\prime }$ is the target Actor network. The Critic network is updated by minimizing the mean squared error loss between the estimated Q-value and the target:

(29) $$L=\frac{1}{N}\sum_{i}{\left(y_{i}-Q\left(s_{i},a_{i}|\theta^{Q}\right)\right)}^{2}$$

The Actor network is updated using the policy gradient, computed as:

(30) $$\nabla_{\theta^{\mu }}J\approx \frac{1}{N}\sum_{i}\nabla_{a}Q\left(s,a|\theta^{Q}\right){|}_{s=s_{i},a=\mu \left(s_{i}\right)}\cdot \nabla_{\theta^{\mu }}\mu \left(s|\theta^{\mu }\right){|}_{s=s_{i}}$$

Here, $\nabla_{\theta^{\mu }}\mu \left(s|\theta^{\mu }\right)$ denotes the gradient of the Actor network’s output action with respect to its parameters, and $\nabla_{a}Q\left(s,a|\theta^{Q}\right)$ is the gradient of the Critic network’s output Q-value with respect to the action input.

To further improve training stability, DDPG employs soft updates to gradually adjust the target network parameters as follows:

(31) $$\begin{array}{cc} \theta^{Q^{\prime }} & \leftarrow \tau \theta^{Q}+\left(1-\tau \right)\theta^{Q^{\prime }},\\ \theta^{\mu^{\prime }} & \leftarrow \tau \theta^{\mu }+\left(1-\tau \right)\theta^{\mu^{\prime }} \end{array}$$

where τ is a small positive constant that ensures smooth target updates. This mechanism helps DDPG maintain structural consistency while improving stability during training.

## 3. Hardware of Robot System

### 3.1. Robot Structure

**Figure 5:**
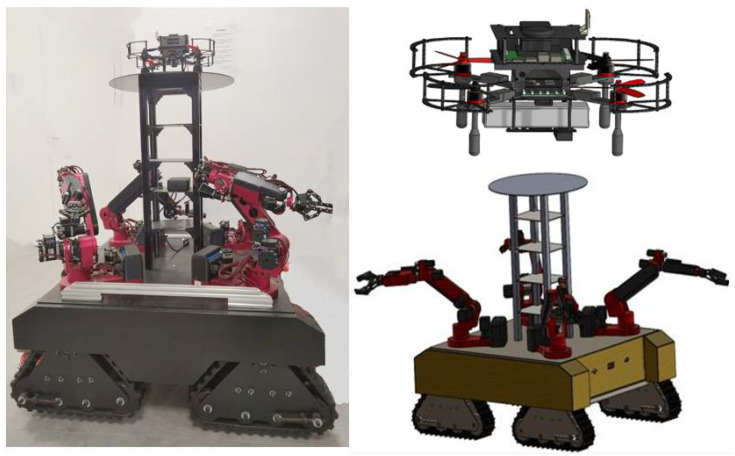
Structure of air–ground collaborative multi-heterogeneous robots.

The structural physical diagram and 3D model of the air–ground collaborative multi-heterogeneous robot system are shown in Figure 5. In disaster rescue scenarios, conventional wheeled or tracked UGVs often perform poorly in soft soil, mud, and narrow spaces. The combination of triangular tracks and differential wheels enhances ground contact, improving traction, stability, and maneuverability, enabling UGVs to effectively navigate complex terrains and perform tasks such as environmental mapping, material transport, and search and rescue [18].

For tasks like rubble search and rescue and obstacle clearance, the four-arm system equipped on the UGV allows for simultaneous operations, including grasping, transporting, cutting, and dismantling, significantly improving operational efficiency. The system’s ability to perform symmetric or complementary movements enhances stability and precision, particularly in confined or irregular spaces [23]. In air–ground collaboration, the UAV landing pad provides essential functions such as takeoff, landing, charging, and maintenance, ensuring stable and efficient operation of unmanned systems.

The 3D model is then imported into the Gazebo simulation environment [24]. Initially, the assembly is exported from SolidWorks as a URDF (unified robot description format) file using an ROS plugin, such as the SolidWorks to URDF plugin. It is essential to ensure that all parts and assemblies are correctly converted into the URDF format, including joints, links, and sensors. The exported URDF file, along with the associated mesh files (such as in STL format), is then placed in the Gazebo model directory. Finally, the URDF file is loaded into Gazebo, completing the configuration of the simulation environment. In the Gazebo simulation environment, the integration of the Mid360 LiDAR and IMU sensors involves configuring the LiDAR sensor within the robot model’s SDF or URDF file. The <sensor> tag is used to define the scanning range, resolution, and point cloud data publishing topic. The IMU sensor is configured using the <sensortype="imu" > tag to specify the accelerometer and gyroscope parameters, along with the IMU data topic. The sensor plugins are activated via the gazebo_ros_pkgs package, and parameter adjustments are made in the ROS configuration files to ensure correct publishing of both LiDAR point cloud and IMU data.

In FAST-LIO2, the data from the IMU is tightly coupled with the LiDAR data to compensate for errors caused by dynamic motion, particularly during rapid movement and rotation. The IMU provides crucial attitude and velocity data that significantly enhance localization accuracy. Additionally, odometry estimates the robot’s displacement and motion by using wheel or track rotation data, typically integrated with IMU and LiDAR data to improve localization precision and path planning reliability. Therefore, the air–ground collaborative multi-heterogeneous robotic system is equipped with sensors such as the MID-360 LiDAR, IMU, and odometry, among others. The MID-360 LiDAR offers distinct advantages over other LiDAR systems, primarily due to its $360^{{}^{\circ}}$ panoramic scanning capability, which provides comprehensive environmental awareness without the need for mechanical rotation. It offers high ranging accuracy and resolution, making it well-suited for precise map construction and obstacle detection in complex environments.

### 3.2. LiDAR Measurement Model

The basic working principle of a LiDAR system involves emitting a laser beam, which reflects off the surface of an object and returns to the LiDAR receiver. By relating the speed of light to the signal propagation time, the distance between the LiDAR and the object can be calculated. This distance is given by:

(32) $$r=\frac{ct}{2}$$

LiDAR acquires 3D point cloud data by scanning both the horizontal and vertical planes, which can be expressed by:

(33) $$\begin{array}{cc} x(\theta ,\phi ) & =r(\theta ,\phi )\cdot \cos\left(\theta \right)\cdot \cos\left(\phi \right)\\ y(\theta ,\phi ) & =r(\theta ,\phi )\cdot \sin\left(\theta \right)\cdot \cos\left(\phi \right)\\ z(\theta ,\phi ) & =r(\theta ,\phi )\cdot \sin\left(\phi \right) \end{array}$$

where θ is the horizontal scanning angle, ϕ is the vertical scanning angle, r(θ,ϕ) is the measurement distance of LiDAT at these two angles.

Let us consider ${\left(x,y\right)}^{T}$ to be the coordinates of the scanned points in the global coordinate system, ${\left(x_{1},y_{1}\right)}^{T}$ is the coordinates of the robot in the global coordinate system, $\theta_{1,2}$ is the rotation angle of the robot in the global coordinate system, ${\left(x_{2},y_{2}\right)}^{T}$ is the installation position of the LiDAR relative to the robot, *z* is the distance of the points measured by the LiDAR. ${\left(x,y\right)}^{T}$ can be expressed as:

(34) $$\left[\begin{array}{c} x\\ y \end{array}\right]=\left[\begin{array}{c} x_{1}\\ y_{1} \end{array}\right]+\left[\begin{array}{cc} \cos\theta_{1,2} & -\sin\theta_{1,2}\\ \sin\theta_{1,2} & \cos\theta_{1,2} \end{array}\right]\left[\begin{array}{c} x_{2}\\ y_{2} \end{array}\right]+z\left[\begin{array}{c} \cos\theta +\theta_{1,2}\\ \sin\theta +\theta_{1,2} \end{array}\right]$$

### 3.3. IMU Measurement Model

The IMU employs triaxial accelerometers and gyroscopes to capture motion parameters of objects. Accelerometers quantify linear acceleration along the X-, Y-, and Z-axes, while gyroscopes measure angular velocities about these axes. By integrating these multi-source kinematic measurements temporally, the IMU derives real-time estimations of the object’s 3D orientation, translational velocity, and positional displacement.

At time instant t, the IMU-estimated 3D coordinates are expressed as $\left(x_{t},y_{t},z_{t}\right)$, accompanied by an attitude angle $\alpha_{t}$, the corresponding translational velocities and accelerations along the orthogonal axes are respectively characterized by $\left\{v_{t}^{x},v_{t}^{y},v_{t}^{z}\right\}$ and $\left\{a_{t}^{x},a_{t}^{y},a_{t}^{z}\right\}$, the angular acceleration of the Euler angles is denoted as $\omega_{t}$. Given the sufficiently short sampling interval, both angular velocity and angular acceleration remain approximately constant over the interval. The velocity and orientation angles at t+Δt are therefore derived as:

(35) $$\begin{array}{cc} v_{t+\Delta t}^{x} & =v_{t}^{x}+a_{t+\Delta t}^{x}\cdot \Delta t\\ v_{t+\Delta t}^{y} & =v_{t}^{y}+a_{t+\Delta t}^{y}\cdot \Delta t\\ v_{t+\Delta t}^{z} & =v_{t}^{z}+a_{t+\Delta t}^{z}\cdot \Delta t\\ \alpha_{t+\Delta t} & =v_{t}+\omega_{t+\Delta t}^{x}\cdot \Delta t \end{array}$$

The post at time t+1 can be expressed by integrating against the above equation:

(36) $$\begin{array}{cc} x_{t+\Delta t} & =x_{t}+v_{t+\Delta t}^{x}\cdot \Delta t\\ y_{t+\Delta t} & =y_{t}+v_{t+\Delta t}^{y}\cdot \Delta t\\ z_{t+\Delta t} & =z_{t}+v_{t+\Delta t}^{z}\cdot \Delta t\\ \alpha_{t+\Delta t} & =\alpha_{t}+\omega_{t+\Delta t}\cdot \Delta t \end{array}$$

Motion distortion in LiDAR data is estimated and compensated through inertial measurements (accelerations and angular rates) provided by the IMU, with implementation specifics comprehensively addressed in the FAST-LIO2 framework section.

## 4. Experiment and Implementation Results

**Figure 6:**
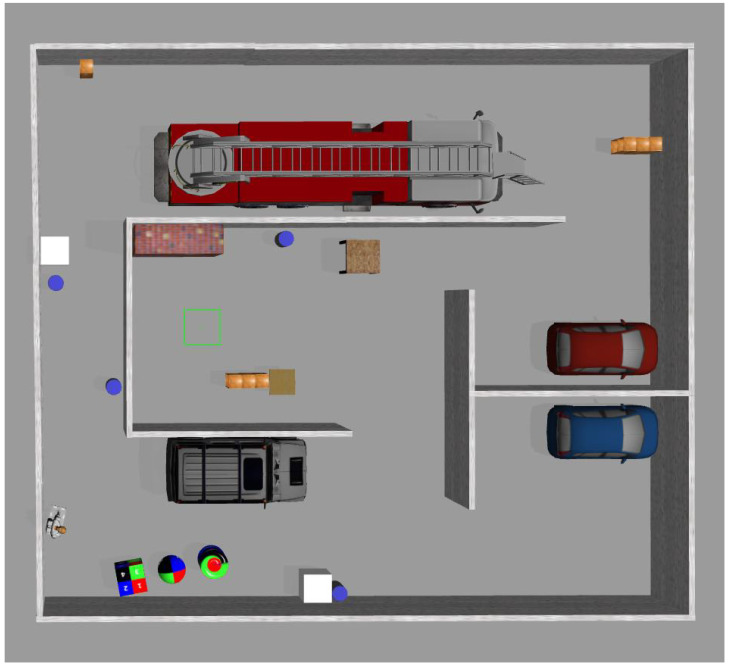
Gazebo simulation environment map.

To simulate the complex environments encountered during disaster relief operations, a simulation map was constructed in Gazebo, as shown in Figure 6, which includes vehicles, walls, and various other obstacles. By parsing the Gazebo world file to extract all the model information, we first obtain the two-dimensional information from the ground plane, and multiply it by the effective height of the world to obtain the total volume of the world. Then, all the models were traversed, and the volume of each obstacle was calculated and accumulated to obtain the total volume of all obstacles. Finally, the obstacle distribution density can be obtained, which is the ratio of the volume of the obstacles to the total volume of the world. Through the above methods, we calculated that the density of obstacles is 17%.

### 4.1. Localization and Mapping of FAST-LIO2

**Figure 7:**
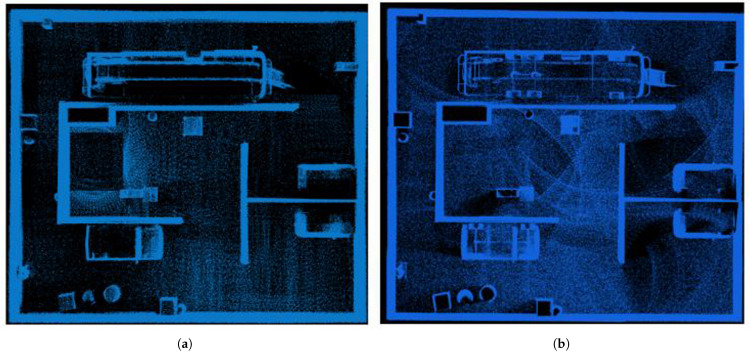
Map construction using FAST-LIO2 in the Gazebo simulation environment: (**a**) result from the UAV; (**b**) result from the UGV.

The UAV was controlled via qgc, while the UGV was operated using rqt_robot_steering, enabling the construction of a 3D point cloud map. Additionally, the trajectory of the UGV was evaluated using evo based on recorded ROS topics. Figure 7 presents the mapping results obtained by FAST-LIO2 for both the UAV and UGV. As shown in the figure, the UAV demonstrates a superior mapping capability for higher regions in the simulated environment, such as the top of the fire truck, while the UGV provides more accurate mapping of lower regions, such as the underside of the fire truck. By fusing the mapping results from both platforms, a more complete and accurate point cloud map can be obtained.

**Figure 8:**
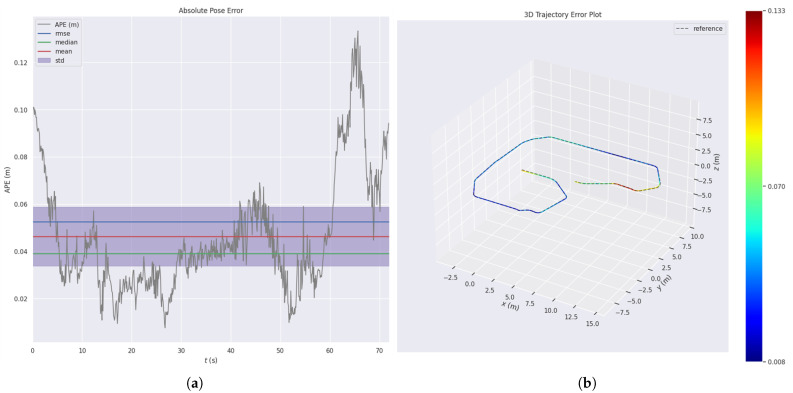
Evo evaluation: (**a**) APE over time; (**b**) 3D trajectory colored by APE.

The absolute pose error (APE) over time and the 3D trajectory colored by APE are illustrated in Figure 8. The APE over time plot presents the temporal evolution of the absolute pose error, along with visual indicators of its mean, median, root mean square error (RMSE), and standard deviation. The 3D trajectory plot visualizes the spatial distribution of APE by encoding the error magnitude at each trajectory point using a color gradient, facilitating intuitive analysis of localization accuracy in space. During the initial phase (0 s∼10 s), the APE exceeds 0.1 m, which is primarily attributed to instability during system initialization and insufficient feature matching in the early stage. In the middle phase (10 s∼55 s), the error stabilizes, with the APE consistently within 0.07 m, indicating high accuracy. The relatively narrow standard deviation range suggests minimal fluctuation, and the system remains consistently stable during this period. In the final phase (55 s∼70 s), the APE increases significantly and reaches its peak, likely due to rapid motion during turning and accumulation of drift, resulting in higher localization errors. Overall, the FAST-LIO2 framework demonstrates high localization accuracy and is suitable for deployment in complex environments.

### 4.2. Fusion of Point Cloud Maps

#### 4.2.1. Evaluation of Preprocess

**Figure 9:**
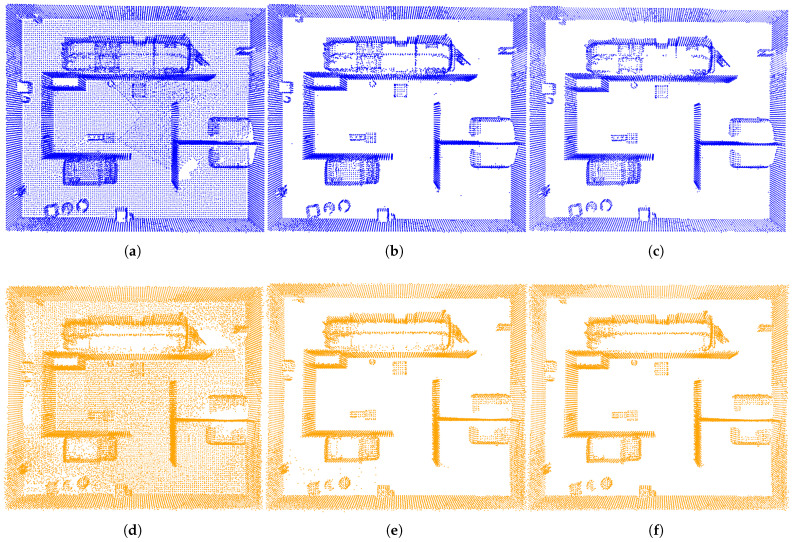
The results of preprocessing. (**a**)–(**c**), (**d**)–(**f**) The outcomes of downsampling, ground segmentation, and filtering for the unmanned ground vehicle and unmanned aerial vehicle in sequence.

The purpose of preprocessing is to enhance data quality, optimize data structure, and establish a more efficient and reliable foundation for subsequent registration tasks. The three preprocessing steps, including downsampling, ground segmentation, and statistical filtering, are illustrated in Figure 9. As shown in the figure, the point cloud density is significantly reduced while key features are preserved. Additionally, ground points and noise have been effectively removed, thereby providing high-quality input for the registration process.

#### 4.2.2. Coarse Registration

**Figure 10:**
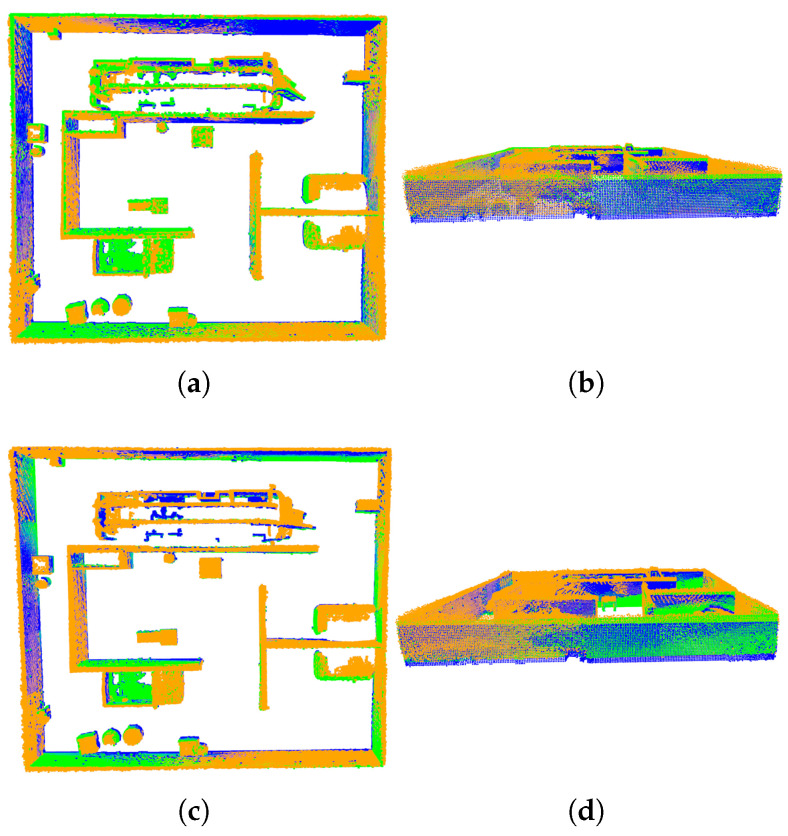
Coarse registration results of the two methods: (**a**,**b**) conventional SAC-IA algorithm; (**c**,**d**) improved SAC-IA algorithm.

In the coarse registration experiments, both the improved SAC-IA algorithm and the conventional SAC-IA algorithm were applied and compared. The results of the coarse registration obtained by both algorithms are shown in Figure 10, where the orange point cloud represents the target point cloud, the blue point cloud represents the source point cloud, and the green point cloud represents the transformed source point cloud. As depicted in the figure, the transformed source point cloud obtained using the improved SAC-IA exhibits a slightly higher overlap with the target point cloud compared to the conventional SAC-IA algorithm. Furthermore, the mean error of the improved SAC-IA algorithm is 0.0422 m, whereas the mean error of the conventional SAC-IA algorithm is 0.0474 m. This indicates that the improved SAC-IA algorithm provides slightly higher registration accuracy than the conventional SAC-IA algorithm.

**Figure 11:**
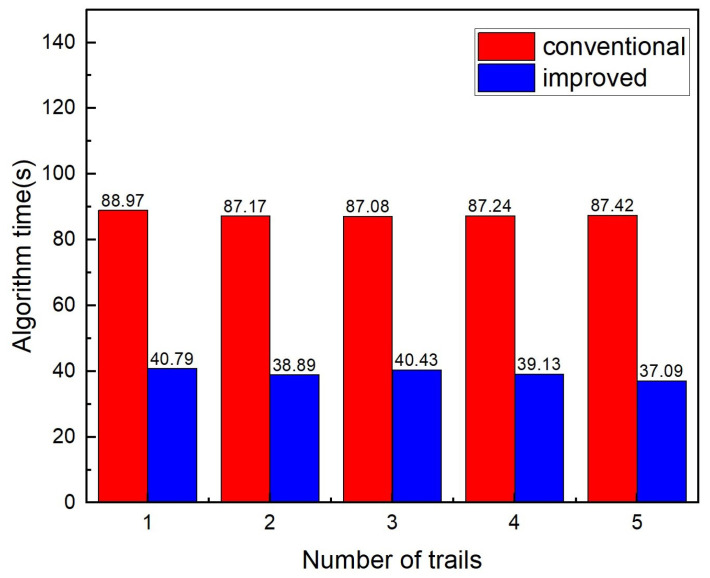
Comparison of computation time between the improved and conventional SAC-IA algorithms.

The time consumption of each algorithm over five runs is shown in Figure 11. The conventional SAC-IA algorithm has an average execution time of 46.25 s, while the improved SAC-IA algorithm achieves an average time of 21.25 s, representing a reduction of over 50%. This demonstrates a significant decrease in execution time, greatly enhancing efficiency, while simultaneously improving the accuracy of coarse registration.

#### 4.2.3. Fine Registration

**Figure 12:**
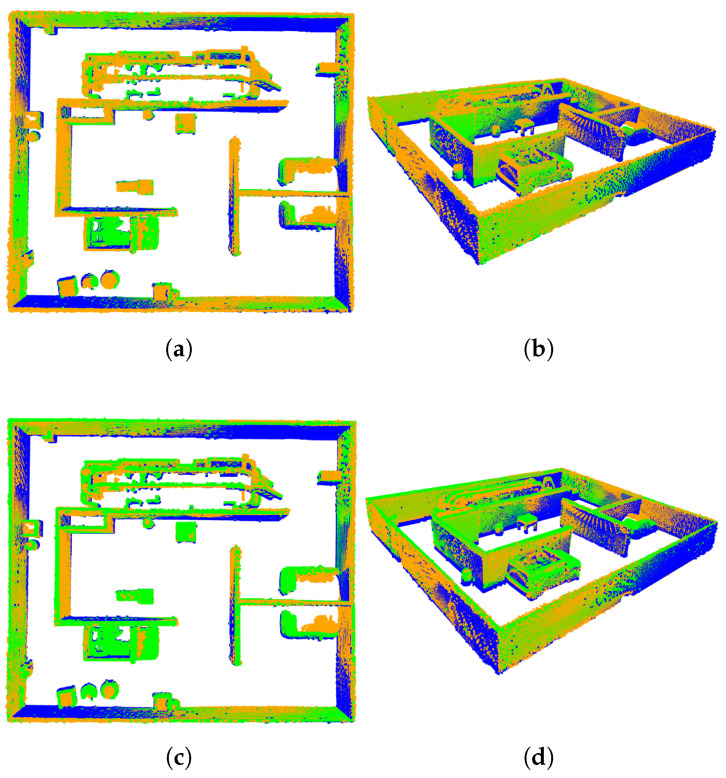
Registration results of different algorithms: (**a**,**b**) are the results after GICP registration, while (**c**,**d**) are the results after VGICP registration.

**Figure 13:**
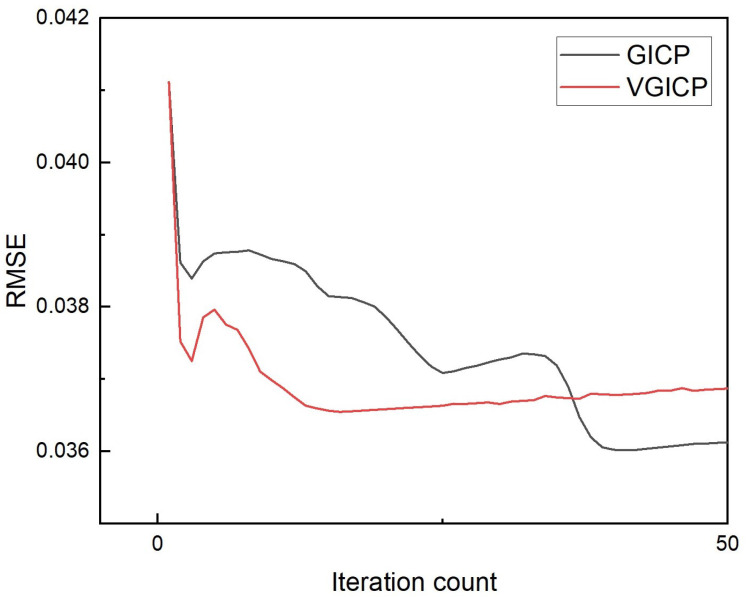
The error convergence curves of GICP and VGICP.

In this experiment, the GICP and VGICP algorithms were employed to perform fine registration and fusion of two point cloud maps obtained through coarse registration. The results of the fusion are shown in Figure 12, with the corresponding fusion time and root mean square error (RMSE) presented in Table 1. In Figure 12, the target point cloud is depicted in orange, the source point cloud in blue, and the transformed source point cloud in green. From the overlap between the target point cloud and the transformed source point cloud in Figure 12, as well as the registration time and RMSE values listed in Table 1, we can know that the registration accuracy of GICP is almost the same as that of VGICP, while the registration speed of VGICP is significantly faster than that of GICP. Theoretically, the registration accuracy of the GICP algorithm is slightly higher than that of VGICP because the more complex optimization model can more accurately consider the geometric relationships and deformation information between point clouds, thereby improving the registration accuracy. However, this improvement in accuracy is usually accompanied by higher computational complexity, resulting in a slower registration speed for GICP. In contrast, VGICP simplifies the registration process through the voxel method, reducing the computational load and the number of iterations, thus achieving a faster registration. Therefore, in general circumstances, VGICP has a faster registration speed compared to GICP while still maintaining a high registration accuracy. The error convergence curves of GICP and VGICP are shown in Figure 13. From the figure, it can be seen that the error convergence speed of VGICP is faster than that of GICP, but GICP has the smallest error, which is consistent with the results in Table 1.

#### 4.2.4. Reinforcement Learning for Path Planning

**Figure 14:**
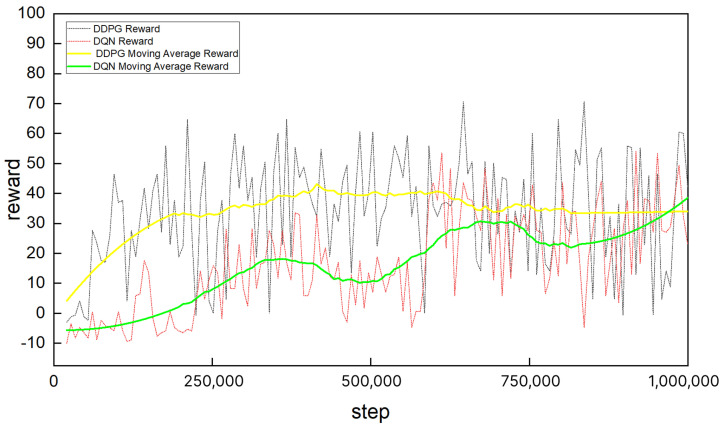
Reward curves of DDPG and DQN.

During the experiment, the reward functions of DDPG and DQN both adopted a piecewise sparse reward function. When the agent reached the goal, the reward value was 80. When the agent collided with an obstacle, our penalty value was −10. The advantage of this approach lies in the clear goal and efficient calculation. By using high rewards and immediate penalties, it clearly distinguishes “reaching the goal” from “colliding”, which meets the core requirements of the path planning task. At the same time, the sparse reward design (with a reward of 0 for the normal steps) reduces redundant signals and improves learning efficiency. The robot’s cumulative training reward curve is shown in Figure 14. The yellow curve represents the moving average reward of DDPG, while the green curve represents that of DQN. As observed from the figure, the DDPG reward curve converges faster than that of DQN. This is attributed to the fact that DQN relies on a discrete action space and an ϵ-greedy exploration strategy, which leads to slower convergence in complex tasks. In contrast, DDPG utilizes a continuous action space and policy gradient optimization, enabling more refined and continuous updates, resulting in faster convergence.

Furthermore, the stability of the DDPG reward curve is superior to that of DQN. This can be explained by the fact that the exploration and environmental randomness of DQN cause significant fluctuations in the reward curve, particularly during the early stages of training. On the other hand, the continuous action selection and stable policy optimization of DDPG lead to a smoother reward curve, with reduced volatility during training and more stable overall performance.

**Figure 15:**
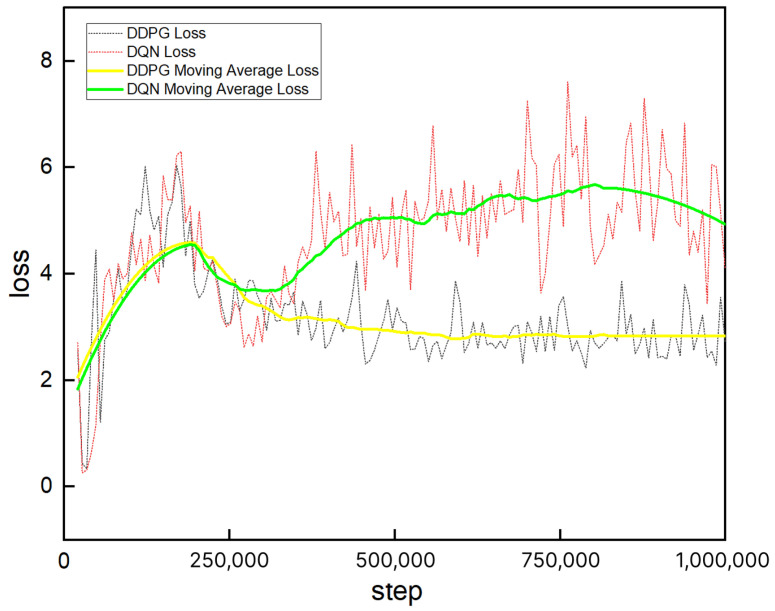
Loss curves of DDPG and DQN.

The training loss curves of the robot are presented in Figure 15, where the yellow curve corresponds to DDPG and the green curve to DQN. It is evident that the DDPG loss curve exhibits greater stability and faster convergence. This is primarily due to the discrete action space and potential estimation errors in the Q-value updates in DQN, which often lead to instability in the loss function and slower convergence. In contrast, DDPG employs a continuous action space and policy gradient optimization, enabling more precise policy adjustments and thereby achieving more stable and efficient convergence.

**Figure 16:**
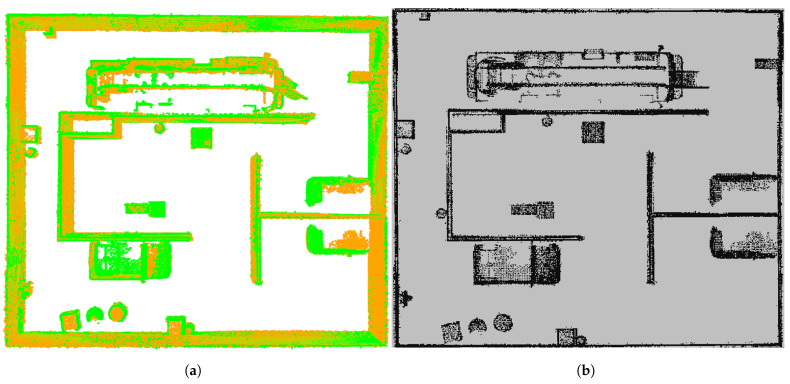
The fused point cloud map (**a**) and its corresponding grid map (**b**).

**Figure 17:**
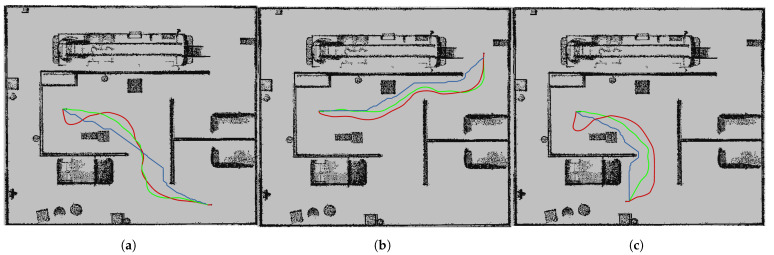
Path planing of DDPG, DQN, and A* algorithms (the green path represents the path planning using DDPG, the red path represents the path planning using DQN, and the blue path represents the path planning using A*. The destinations in Figure (**a**–**c**) are labeled A, B, and C).

The point cloud map after fine registration and its corresponding grid map are shown in Figure 16. In this experiment, path planning using DQN, DDPG, and A* algorithms is performed on the grid map. The path planning results for these three algorithms are shown in Figure 17, where each algorithm plans paths to three different destinations. The total path lengths for each algorithm to reach the different destinations are summarized in Table 2.

From Table 2, it can be observed that the DDPG path planning algorithm outperforms the DQN algorithm in terms of both path length and smoothness. This is due to DDPG’s use of a continuous action space, whereas DQN relies on a discrete action space. DDPG allows for fine-grained adjustments of the robot’s movement at each time step, enabling precise control over steering angles and speeds, thereby preventing abrupt changes and irregularities in the path, resulting in smoother trajectories. Additionally, the continuous action space of DDPG provides greater flexibility in obstacle avoidance, reducing the overall path length. In contrast, DQN’s discrete action selection may lead to longer and less smooth paths. Consequently, DDPG achieves more efficient path planning through finer control and strategy optimization, leading to shorter and smoother paths.

Although the A* algorithm yields the shortest path, it is based on a discrete action space, limiting the precise control of path details, resulting in paths that are not smooth and lack flexibility [25]. Furthermore, A* assumes a static environment, making it unable to adapt to changes in dynamic environments. When the environment changes, A* requires re-planning, whereas DDPG can continuously adjust its strategy in real-time to adapt to such changes.

## 5. Conclusions and Future Works

This paper addresses the key challenges of robot mapping and autonomous navigation in disaster rescue scenarios by proposing a collaborative air–ground heterogeneous multi-robot system framework. By leveraging the strengths of UAV and UGV, the system enables efficient environmental perception and path planning. Experimental results demonstrate that the FAST-LIO2 localization error (RMSE) consistently remains within 0.07 m during stable operation, maintaining high stability even in complex motion scenarios, thus proving its practical value in disaster rescue missions. The efficient fusion of point cloud maps is achieved using an improved SAC-IA coarse registration algorithm and the VGICP fine registration algorithm. Experiments show that the improved SAC-IA algorithm significantly outperforms traditional methods in both registration accuracy and computational efficiency (with average error reduced by 10.9% and average time consumption reduced by 54%). Meanwhile, VGICP enhances the registration speed by 39% while maintaining accuracy, providing a reliable solution for real-time map construction in complex disaster environments. Compared to traditional DQN and A* algorithms, the DDPG algorithm excels in continuous action spaces, generating smoother and shorter trajectory paths.

The current experiment is based on a simulated environment, and future work will focus on testing the system’s robustness against interference in real-world complex scenarios such as rubble, landslides, and other disaster environments, particularly optimizing its robustness to sensor noise, extreme lighting conditions, and dynamic obstacles. The training time of the DDPG algorithm is relatively long and relies on a large amount of interaction data. Future efforts may incorporate transfer learning or meta-learning strategies to enhance the algorithm’s generalization capability across different disaster scenarios. Additionally, exploring a multi-agent collaborative reinforcement learning framework could optimize task allocation and collaborative path planning between air and ground robots.

## Figures and Tables

**Table 1:** Registration time and accuracy. The registration time is the average of five runs, and the RMSE is calculated based on the point cloud data after registration.

	Registration Time	RMES
GICP	0.3737	0.0360
VGICP	0.2270	0.0365

**Table 2:** Distance to different destinations for different algorithms.

Algorithm	Destination A	Destination B	Destination C
DDPG	22.74	20.90	19.80
DQN	21.36	21.13	19.86
A*	19.76	15.10	14.41

## Data Availability

The data presented in this study are available on request from the corresponding author. The data are not publicly available due to privacy or ethical restrictions.
